# A stretchable, electroconductive tissue adhesive for the treatment of neural injury

**DOI:** 10.1002/btm2.10667

**Published:** 2024-05-03

**Authors:** Jharana Dhal, Mahsa Ghovvati, Avijit Baidya, Ronak Afshari, Curtis L. Cetrulo, Reza Abdi, Nasim Annabi

**Affiliations:** ^1^ Department of Chemical and Biomolecular Engineering University of California – Los Angeles Los Angeles California USA; ^2^ Department of Radiological Sciences David Geffen School of Medicine, University of California – Los Angeles Los Angeles California USA; ^3^ Division of Plastic Surgery Massachusetts General Hospital Boston Massachusetts USA; ^4^ Transplantation Research Center, Nephrology Division Brigham and Women's Hospital Boston Massachusetts USA; ^5^ Department of Bioengineering University of California – Los Angeles Los Angeles California USA

**Keywords:** bioadhesive, bioionic liquid, elastin‐like polypeptide, electroconductivity, nerve repair

## Abstract

Successful nerve repair using bioadhesive hydrogels demands minimizing tissue–material interfacial mechanical mismatch to reduce immune responses and scar tissue formation. Furthermore, it is crucial to maintain the bioelectrical stimulation‐mediated cell‐signaling mechanism to overcome communication barriers within injured nerve tissues. Therefore, engineering bioadhesives for neural tissue regeneration necessitates the integration of electroconductive properties with tissue‐like biomechanics. In this study, we propose a stretchable bioadhesive based on a custom‐designed chemically modified elastin‐like polypeptides (ELPs) and a choline‐based bioionic liquid (Bio‐IL), providing an electroconductive microenvironment to reconnect damaged nerve tissue. The stretchability akin to native neural tissue was achieved by incorporating hydrophobic ELP pockets, and a robust tissue adhesion was obtained due to multi‐mode tissue–material interactions through covalent and noncovalent bonding at the tissue interface. Adhesion tests revealed adhesive strength ~10 times higher than commercially available tissue adhesive, Evicel®. Furthermore, the engineered hydrogel supported in vitro viability and proliferation of human glial cells. We also evaluated the biodegradability and biocompatibility of the engineered bioadhesive in vivo using a rat subcutaneous implantation model, which demonstrated facile tissue infiltration and minimal immune response. The outlined functionalities empower the engineered elastic and electroconductive adhesive hydrogel to effectively enable sutureless surgical sealing of neural injuries and promote tissue regeneration.

**Translational Impact Statement**

Our research introduces a groundbreaking stretchable adhesive hydrogel designed to reconnect damaged nerve tissues and seal injuries without the need for stitches. Our designed hydrogel is composed of biocompatible materials with mechanical properties inspired by native neural tissues. This hydrogel outperforms current commercial sealants in adhesion while showing electroconductivity, aiding in the transmission of neuro signals to support normal nerve function. With promising mechanical, conductivity, biocompatibility, and biodegradability results, our discovery has the potential to transform nerve injury treatment, bringing us closer to sutureless and effective healing.

## 1 INTRODUCTION

Traditionally, treatment of peripheral nerve injuries involves microsurgical suturing, which often leads to increased inflammation and scar tissue formation at the injury site. Such surgical procedures also raise the risk of permanent tissue damage and result in poor functional recovery. Statistically, less than half of patients who undergo traditional treatment procedures for nerve repair regain full functionality.^1^, ^2^ In the case of larger nerve injuries requiring a nerve graft, the recovery rate drops below 25% due to the involvement of two coaptation sites.^1^ Current clinical treatments involving end‐to‐end suturing of nerve bundles significantly induce intraneural inflammation, secondary damage, and fibrosis. A recent report indicates that nearly 33% of cases with peripheral nerve injuries treated at clinics showed loss or partial recovery of motor and sensory function, chronic pain, and end‐target muscle atrophy.^3^ Consequently, for the treatment of soft nerve tissue, the introduction of a minimally invasive surgical procedure, that employs an adhesive biomaterial to avoid suturing injured or ruptured neural tissue bundles, could potentially become the gold standard in clinical settings.

Recently, few bioadhesive materials based on fibrin (e.g., Evicel®, TISSEEL™) and cyanoacrylate (e.g., Dermabond™) have been commercially available for clinical treatments of nerve injuries. However, in terms of functional recovery, fibrin‐based glues have demonstrated mixed results.^4^, ^5^, ^6^, ^7^, ^8^ For instance, while fibrin‐based glues have shown biocompatibility, they lack tissue‐mimicking mechanical integrity and rigidity, with lower elasticity and stiffness compared to native neural tissue. They also increase the risk of infectious disease transmission.^9^, ^10^ Meanwhile, cyanoacrylate‐based adhesives have shown serious concern due to their cytotoxicity and inflammatory response toward nerve tissue.^11^, ^12^ Moreover, most of these adhesives need to be applied in conjunction with the suturing of the tissues. Hence, as an alternative to these commercial glues, there is a need for an elastic, biodegradable, biocompatible, and mechanically stable adhesive material that can be applied over wet injury sites to offer effective sutureless treatment. This material should also mimic nerve tissue elasticity while promoting tissue regeneration.

Functionally neural tissues, such as the central nervous system (CNS) and peripheral nervous system (PNS), mediate information along nerve fibers in the form of electrical impulses. Endogenous bioelectric signals in the spinal cord sustain neuronal function, neurite growth, and nerve regeneration.^13^, ^14^, ^15^, ^16^, ^17^ Therefore, the introduction of electrical stimulation is essential for treating nerve injuries and expediting peripheral nerve regeneration.^18^ Additionally, similar to the application of electrical stimulation, nerve cells have demonstrated sensitivity to the conductive physiochemical microenvironment and have exhibited enhanced growth and differentiation when seeded on conductive substrates.^17^, ^19^, ^20^, ^21^, ^22^

Notably, the elastic nature of materials has also been shown to influence the growth and function of nerve cells.^23^, ^24^ For instance, Jiang et al. demonstrated that elastic modulus can affect neural stem cell differentiation into various nerve cells.^25^ In addition, reducing mechanical mismatch across the material–tissue interface substantially minimizes adverse immune responses during chronic implantation and scar tissue formation.^26^, ^27^, ^28^ Thus, an unmet need exists to engineer an electroconductive elastic bioadhesive hydrogel for soft neural tissue repair capable of mimicking the mechano‐physical properties of native nerve tissue to enhance neuro‐regeneration and functional recovery.

Over the past few decades, a wide range of conductive agents have been incorporated into biomaterials for the treatment of nerve tissue injuries.^29^, ^30^, ^31^, ^32^ This includes synthetic conductive polymers such as polypyrrole (PPy), polyaniline (PANI), and poly(3,4‐ethylenedioxythiophene) (PEDOT), or carbon‐based materials such as carbon nanotubes (CNTs) and graphene oxide (GO).^33^, ^34^, ^35^, ^36^ Although these materials provide physiologically relevant conductivity, they inherently possess some disadvantages. For example, the incorporation of conductive polymers, such as PEDOT, PANI, and PPy into scaffolds can reduce their biodegradability and biocompatibility.^37^ Some studies have also reported that CNTs and GO can elicit toxic effects in living systems, which may increase with their in concentration and exposure time.^38^ Additionally, the large surface area of these nanomaterials can cause the production of reactive oxygen species and antioxidant deactivation.^39^

Recently, we designed a gelatin‐based conductive and adhesive hydrogel that showed low immunogenicity when implanted subcutaneously in rats.^40^ However, the lack of desired elasticity similar to the native neural tissue may restrict its application in the repair and sealing of nerve injuries. Meanwhile, our recent study based on gelatin methacryloyl (GelMA) and methacryloyl substituted tropoelastin (MeTro)‐based bioadhesive hydrogels demonstrated tunable mechanical properties that could be suitable for nerve repair. However, the engineered hydrogel failed to provide an electroconductive microenvironment for enhanced neural tissue regeneration.^41^

To address these limitations, in this study we engineer a highly stretchable, biodegradable, biocompatible, and conductive bioadhesive that mimics the mechano‐chemical properties and microenvironment of nerve tissues, facilitating the sealing and repair of neural injuries. To achieve the desired mechanical properties of soft and stretchable nerve tissue, a custom‐designed elastin‐like polypeptide (ELP) sequence was first chemically functionalized with methacrylate groups. We hypothesize that methacrylated ELP (mELP), with hydrophobic units, would enable reversible molecular deformation and imparts elastic characteristics to the engineered hydrogel.^42^ To introduce conductivity, an acrylated choline‐based bioionic liquid (Bio‐IL) with a positive charge‐ended molecular structure, was combined with the mELP to provide ionic conductivity to the resulting mELP/Bio‐IL hydrogel. To demonstrate the clinical applicability of the engineered hydrogel, in vitro cytotoxicity, and in vivo biocompatibility and biodegradability were evaluated using human oligodendrocytic cells (MO3.13), and subcutaneous implantation into the dorsal skin of rats, respectively. The engineered elastic and conductive bioadhesive has the potential for the minimally invasive treatment and repair of conductive soft organs such as nerve tissues.

## 2 MATERIALS AND METHODS

### 2.1 Synthesis of methacrylated elastin‐like polypeptide

ELP was synthesized from genetically engineered kanamycin‐resistant *Escherichia coli* (*E. coli*) and purified using an inverse transition cycle method, as described in our previous article.^42^ Then, 10% (w/v) of the purified ELP was dissolved in 4°C Milli‐Q water and 15% (v/v) methacrylic anhydride (MA, Sigma‐Aldrich) was added dropwise to the solution. The reaction mixture was continuously stirred at 4°C for 16 h. Subsequently, the reaction mixture was diluted with Milli‐Q water (4× volume) and dialyzed in a dialysis cassette against Milli‐Q water, with water changes performed twice daily at 4°C for 5 days. Following dialysis, the purified solution was frozen, lyophilized, and stored in the fridge for further use.

### 2.2 Fabrication of mELP/Bio‐IL hydrogel

To fabricate mELP/Bio‐IL hydrogel, Bio‐IL was first synthesized following the methodology described in our previous work.^40^ Subsequently, a stock solution of Irgacure 2959 (Sigma‐Aldrich) was prepared by dissolving 5% (w/v) Irgacure 2959 in Milli‐Q water and maintained at 65°C. Then, composite precursor solutions of mELP and Bio‐IL were prepared by mixing 15% (w/v) mELP with different concentrations of Bio‐IL in water (7%, 11%, and 15% (v/v)) which were subsequently vortexed (3000 rpm) at 4°C for 2 h until it completely dissolved. Irgacure 2959 stock solution was mixed with polymer mixtures just before photocrosslinking to achieve a final concentration of 0.05% (w/v) Irgacure 2959. Then, prepolymer solutions were cast onto a polydimethylsiloxane (PDMS) mold pre‐cooled to 4°C and photocrosslinked with UV light (6.9 mW/cm^2^, EXFO OmniCure S2000) for 240 s.

### 2.3 ^1^H NMR characterization of mELP and mELP/Bio‐IL hydrogels

Chemically modified mELP was characterized using proton nuclear magnetic resonance (^1^H NMR, 400 MHz Bruker AV400 spectrometer) in deuterated dimethyl sulfoxide (DMSO‐d6, Cambridge Isotope Laboratories, Inc). For quantitative analysis of the degree of methacrylation (DM), an aromatic peak associated with the tryptophan (Trp (W)) residue was utilized as a reference. Then, the area under the peaks related to the methacrylate group and Trp (W) were integrated using MestReNove software. DM was calculated using the following equation (Equation (1)):

(1) $$\mathrm{DM}=\frac{\text{integrated area of methacrylate protons}}{\text{integrated area of reference peak}}\times \text{number of protons in methacrylate group}$$

The degree of crosslinking of mELP/Bio‐IL hydrogel was also calculated using Equation (2), where PA_b_ is the peak area before crosslinking, and PA_a_ is the peak area after crosslinking:

(2) $$\text{degree of crosslinking}\left(\%\right)=\frac{{\mathrm{PA}}_{b}-{\mathrm{PA}}_{a}}{{\mathrm{PA}}_{b}}\times 100$$

### 2.4 Mechanical characterization of mELP/Bio‐IL hydrogels

To characterize the mechanical properties of the engineered hydrogel, the pre‐polymer solutions were pipetted onto rectangular‐shaped PDMS molds pre‐cooled at 4°C and crosslinked with UV light (6.9 mW/cm^2^, EXFO OmniCure S2000) for 240 s. The tensile properties of the hydrogels were measured by conducting tensile tests and cyclic tensile tests on crosslinked thin rectangular samples (12 mm in length, 4.5 m in width, 1 mm in thickness) with a mechanical testing machine (Instron 5943). For the tensile test, the samples were stretched at a rate of 1 mm/min until failure. The tensile strain (%) and tensile stress (kPa) were measured and collected with BlueHill Universal software. The percentage of maximum stretchability was calculated based on the maximum strain achieved before rupture. Elastic modulus was obtained by calculating the slope of the linear region of the stress–strain curves (0–10% strain), while the toughness was calculated as the area under the stress–strain curve.^43^, ^44^ For the cyclic tensile test, the rectangular hydrogel samples were stretched up to 50% strain at a rate of 1 mm/min, and followed by relaxation up to 0% strain at a similar rate (10 cycles). The energy loss percentage was calculated by measuring the difference between the integrals of the stretching and relaxation curves for each cycle.

### 2.5 In vitro swelling properties of mELP/Bio‐IL hydrogels

For the swelling test, rectangular samples, similar to the tensile test, were prepared as described in the previous section. The crosslinked samples were initially weighed (*w*_0_), and then submerged in 1 mL Dulbecco's phosphate‐buffered saline (DPBS), each in separate wells of a 24‐well plate and incubated at 37°C. Subsequent weight measurements (*w*_*t*_) of the samples were taken after 2, 4, 16, and 24 h, and fresh DPBS was added at every interval. The swelling ratio at each time point was calculated as the ratio of the difference in weight of samples weighed at a time interval (*w*_*t*_) and initial weight (*w*_0_) to initial weight (*w*_0_) using Equation (3):

(3) $$\text{swelling ratio}\left(\%\right)=\frac{w_{t}–w_{0}}{w_{0}\;}\times 100$$

### 2.6 Ex vivo adhesive properties of mELP/Bio‐IL hydrogels

A wound closure test (ASTM F2458‐05) was performed to determine the adhesive strength of the engineered hydrogels.^45^, ^46^, ^47^, ^48^, ^49^ Rat peripheral nerve was used as the biological substrate to evaluate the relative adhesion strength of various hydrogel formulations. The tissue was cut into 1 cm‐length pieces and at their intersection points, 70 μL of prepolymer solution was pipetted and crosslinked using UV light for 240 s. Two free ends of the tissue were then attached to glass slides using superglue with a 0.5 cm overhang. The glass slides were then mounted on the Instron 5943 mechanical tester, and tensile loading was conducted at a strain rate of 1 mm/min until failure. The adhesive strength was determined by recording the maximum stress at the point of tissue detachment, as indicated on the stress strain curve.

### 2.7 In vitro electrical conductivity of mELP/Bio‐IL hydrogels

To determine the conductivity of the hydrogels, a two‐probe method of conductivity measurements was employed.^50^ Briefly, mELP/Bio‐IL hydrogels were synthesized following the previously described procedure and connected to two metallic electrodes. The electrodes were positioned on opposing sides of the hydrogel and subsequently connected to a potentiostat (model 263A, AMETEK). A direct current (DC) voltage was applied through the electrodes, ranging from −3 to 3 V in 0.03 V increments. The variation in current with changing voltage was measured, and conductivity was calculated using Ohm's law.

### 2.8 Ex vivo evaluation of electrical conductivity of mELP/Bio‐IL hydrogels

Abdominal tissues from adult female Wistar rats were used to assess the electrical conductivity of the hydrogels ex vivo. Immediately after euthanasia, the rectus abdominus tissue was removed from the rats and placed in DPBS. The rectus abdominus was cut into small pieces (10 mm length) and placed adjacently with a 1 mm gap. Pure mELP and mELP/Bio‐IL prepolymer solutions were then pipetted into the gap between the tissues and photocrosslinked as described previously. Thereafter, electric pulses of 50 ms were applied to one side of the repaired tissue from a voltage source (potentiostat; Agilent, 4284A). By increasing the applied voltage, the threshold voltage required to induce muscle contraction on the adjacent side of the muscle tissue was determined. This measurement was taken as an indicator of successful electrical pulse conduction across the hydrogel‐mediated interface.

### 2.9 In vitro cytotoxicity of mELP/Bio‐IL hydrogels

The in vitro viability and metabolic activity of human oligodendrocytic (Glial; MO3.13) cells were evaluated to check the cytocompatibility of mELP/Bio‐IL hydrogels using transwell permeable supports. Cell viability and proliferation were assessed using a commercial live/dead kit (Invitrogen™) and F‐actin/DAPI staining (Invitrogen™; Millipore Sigma), respectively. In addition, metabolic activity was evaluated using a PrestoBlue assays (Life Technologies). The mELP/Bio‐IL hydrogel (15% (w/v) mELP/15% (v/v) Bio‐IL), and pure mELP, as control, were evaluated for cytocompatibility.

Initially, MO3.13 cells were seeded at the bottom of 24‐well transwell permeable supports. The hydrogel compositions were fabricated in cylindrical shapes (5 mm diameter and 4 mm thickness) using a PDMS mold. Thereafter, the hydrogels were placed into the transwell inserts, and 1 mL of growth medium (DMEM, high glucose (Gibco) supplemented with 10% fetal bovine serum (FBS, Invitrogen™) and 1% penicillin‐streptomycin (Invitrogen™)) was added to each well of the transwell permeable supports. Then, the cell‐seeded well plates were placed and maintained at 37°C in a 5% CO_2_ humidified atmosphere for 5 days and the culture medium was replaced every 48 h.

The viability of MO3.13 cells was assessed using a commercial live/dead viability kit (Invitrogen™), following the manufacturer's instructions. Briefly, cells were stained with 2 μL/mL of ethidium homodimer‐1 (EthD‐1), and 0.5 μL/mL of calcein AM in DPBS (HyClone™) for 30 min at 37°C. Fluorescent imaging was conducted on days 1 and 5 post‐seeding using an AxioObserver Z1 inverted microscope. Live and dead cells were distinguished by green and red color respectively. The number of live and dead cells was quantified using the Image J software, and cell viability (%) was determined by calculating the ratio of the number of live cells to the total number of cells.

The metabolic activity of MO3.13 cells was evaluated at days 1 and 5 post‐seeding using a PrestoBlue assay (Life Technologies). In brief, MO3.13 cell cultures were incubated in 400 μL of growth medium containing 10% (v/v) PrestoBlue reagent for 40 min at 37°C. Then, the fluorescence of the incubated solution was measured using a Synergy HT fluorescence plate reader (BioTek). MO3.13 cells spreading at the bottom of the 24‐well transwell permeable supports were visualized through fluorescent staining of F‐actin (using Alexa Fluor™ 488 Phalloidin (Invitrogen™)) and cell nuclei using DAPI (4′,6‐diamidino‐2‐phenylindole; Millipore Sigma). Briefly, cell cultures at days 1 and 5 post‐seeding were fixed using 4% (v/v) paraformaldehyde (Sigma) for about 15 min. The fixed cells were permeabilized in 0.1% (w/v) Triton X‐100 (Sigma) for 5 min and blocked in 1% (w/v) bovine serum albumin (BSA, Sigma) for 30 min. Then, the samples were incubated with F‐actin for 45 min, followed by three times washing with DPBS. Next, the samples were stained with 1 μL/mL DAPI in DPBS for 1 min. Fluorescent imaging was performed using an AxioObserver Z1 inverted microscope.

### 2.10 In vivo biocompatibility and biodegradability of mELP/Bio‐IL hydrogels

To ensure the suitability of our engineered hydrogels as a surgical sealant, in vivo biocompatibility and biodegradability of mELP and mELP/Bio‐IL hydrogels were assessed using a rat subcutaneous implantation model. The in vivo studies conducted for this research were approved by the Institutional Animal Care and Use Committee (IACUC) at the University of California−Los Angeles (UCLA), under the approval number: 2018–076‐01C. Male Wistar rats (200–250 g) sourced from Charles River Laboratories (Boston, MA, USA) were accommodated in the animal facilities of UCLA. Anesthesia was achieved by inhalation of isoflurane (2–2.5%). After anesthesia, eight incisions of 1 cm were made on the dorsal skin of rats, and small subcutaneous pockets were made using a blunt scissor. Then, mELP/Bio‐IL with 15% (v/v) Bio‐IL and pure mELP hydrogels were fabricated in a cylindrical shape PDMS mold (5 mm in diameter and 4 mm in depth) as described in the previous sections. The hydrogels were sterilized under UV light for 10 min. The sterile hydrogels were then lyophilized, weighted (to have the initial weight for the biodegradation study), and implanted into the subcutaneous pockets and the incisions were closed with 4–0 polypropylene sutures (AD Surgical). At days 7 and 28 post‐implantation, the animals were euthanized and the hydrogels were explanted with and without the surrounding tissues for histological assessment and biodegradation study, respectively. (A total of two rats were used in the study: one was euthanized on day 7 and the other on day 28. For each group, four samples were tested.)

Histological analyses were performed on the explanted hydrogels to investigate the inflammatory responses caused by the implanted hydrogels. After explantation of hydrogel samples with the surrounding tissues, they were fixed with 4% paraformaldehyde for 4 h and incubated in 15% and 30% sucrose at 4°C. Samples were then embedded in an Optimal Cutting Temperature compound (OCT, Fisher Healthcare), frozen in liquid nitrogen, and sectioned using Leica CM1950 cryostat machine. Eight micrometers sections were mounted on positively charged slides using DPX mountant (Sigma) for hematoxylin and eosin (H&E) staining and Masson's Trichrome (MT) staining, and ProLong™ Gold antifade reagent (Thermo Fisher Scientific) for fluorescent immunohistochemistry (IHC) staining. The slides were further processed for H&E and MT staining (Sigma) according to manufacturer instructions. Fluorescent IHC staining was also performed on mounted samples, as previously reported.^41^ Anti‐CD68 (ab125212) (Abcam) was used as the primary antibody, and Goat‐anti Rabbit IgG (H + L) secondary antibody conjugated to Alexa Fluor® 488 (Invitrogen™) was used as a detection reagent. All samples were then stained using DAPI. Lastly, imaging was performed using a ZEISS Axio Observer Z1 inverted microscope.

### 2.11 Statistical analysis

Statistical analysis of all numerical data was carried out using an ANOVA test (one‐way) with GraphPad Prism software. The error bars were evaluated as the mean ± standard deviation (SD) of measurements (*p < 0.05, **p < 0.01, ***p < 0.001, ****p < 0.0001).

## 3 RESULTS AND DISCUSSION

### 3.1 Synthesis and chemical characterization of mELP and Bio‐IL

**FIGURE 1:**
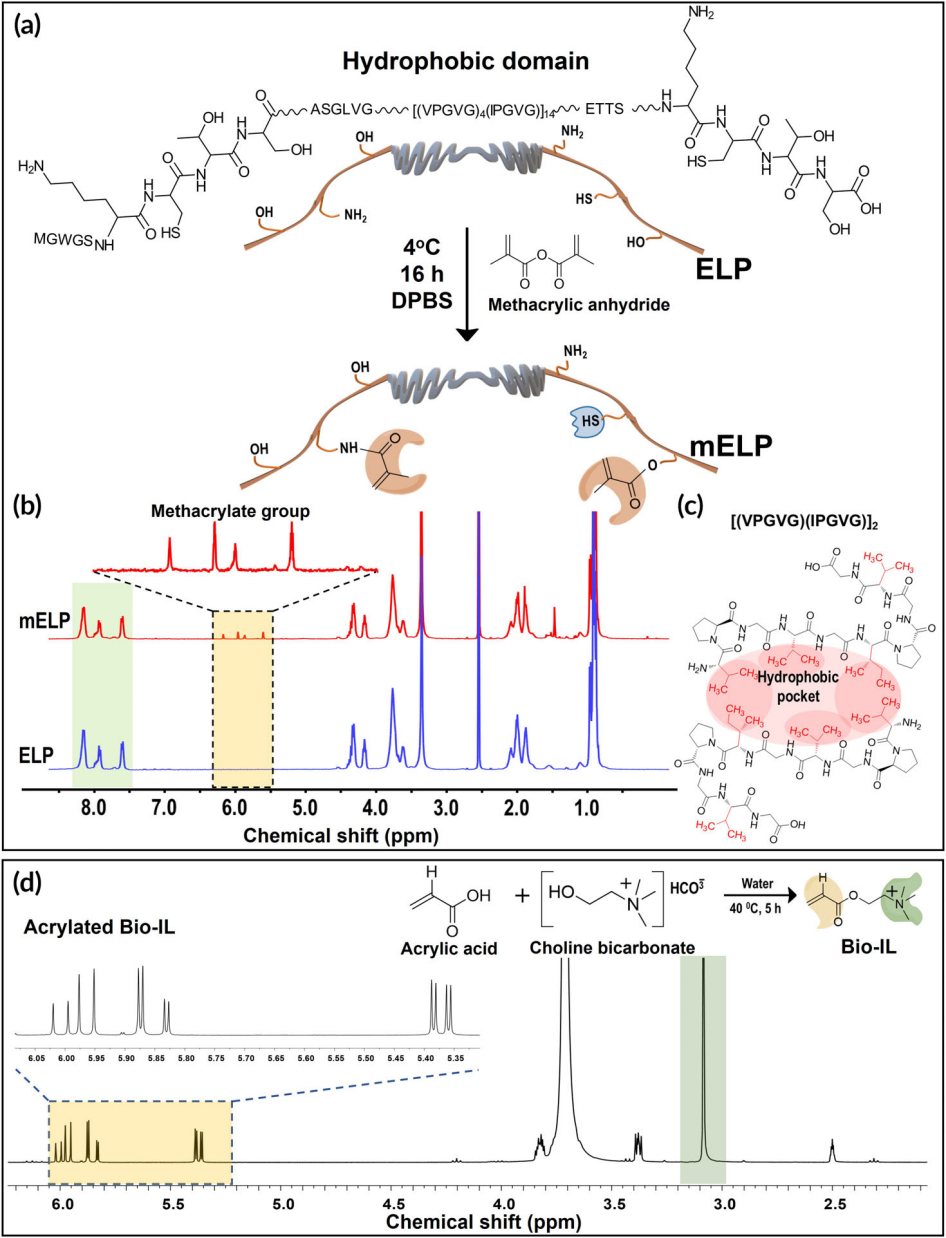
Synthesis and chemical structures of mELP and Bio‐IL. (a) Schematic representation of the chemical structures of ELP and mELP; (b) ^1^H NMR spectra of ELP and mELP; (c) hydrophobic pockets in ELP structure from amino acid residues having hydrophobic residues; (d) synthesis of Bio‐IL from choline bicarbonate and acrylic acid conjugation reaction and corresponding ^1^H NMR spectrum. DPBS, Dulbecco's phosphate‐buffered saline; ^1^H NMR, proton nuclear magnetic resonance; Bio‐IL, acrylated choline‐based bioionic liquid; ELP, elastin‐like polypeptide; mELP, methacrylated ELP.

Elastin‐derived macromolecules, known as ELPs, possess remarkable mechano‐chemical properties due to their sequential amino acid building blocks, which include hydrophobic domains.^42^, ^51^ The genetically encoded design and recombinant synthesis of ELPs enable precise control of their physicochemical properties, which have led to a wide range of biomedical applications. Herein, a custom‐designed ELP macromolecule was synthesized with genetically engineered *E. coli* containing 70‐pentapeptide repetitive units (Figure 1a). These repetitive pentapeptide units, consisting of VPGVG amino acid residues, were modified every five units of isoleucine with valine (([VPGVG]_4_[IPGVG])_14_).^42^ The substitution of isoleucine with valine in this custom‐designed ELP provides tunability in physical properties and functionality, including hydrophobicity, transition temperature, and stability.^52^ Valine possesses distinct hydrophobic and structural characteristics compared to isoleucine, thus influencing the overall solubility of the ELP required for the intended application. Moreover, the VPGVG repetitive units play a critical role in ELP's self‐assembly behavior, biocompatibility, and interaction with biological systems.^53^ Therefore, strategically substituting valine for isoleucine can achieve the desired biological properties. This level of customization is one of the key advantages of using genetically engineered systems for synthesizing ELPs. The synthesized ELP molecule also included a lysine‐cysteine‐threonine‐serine (Lys‐Cys‐Thr‐Ser) sequence containing chemically reactive amine and hydroxyl functionalities (Figure 1a). To engineer a photocrosslinkable hydrogel with tunable mechanical properties, the amine and hydroxyl functionalities of the engineered ELP molecule were covalently conjugated with methacrylate groups, resulting in the formation of mELP (Figure 1a).

For this purpose, we first modified ELP with two different degrees of methacrylation (DM) through varying concentrations of methacrylic anhydride (MA) used to synthesize mELP. mELPs were synthesized with a high degree of methacrylation (DM) using 15% (v/v) MA and with a lower DM using 7.5% (v/v) MA. Verification of successful methacrylation on both mELP backbones was confirmed via ^1^H NMR analysis (Figure 1b and Figure S1). Results showed the emergence of the methacrylate (ɑ/β) and the methacrylamide (γ/δ) proton peaks for mELP with both low and high DM (Figures 1b and S1; highlighted in yellow) in the region of 5.5 to 6.3 ppm. In addition, the presence of distinct ELP peaks (Figure 1b and S1; highlighted in green), within the region of 7.5–8.3, before and after methacrylation confirmed its structural stability during the reaction. For quantitative analysis of the DM, an aromatic peak at 7.5 ppm related to Trp (W) residue was used as a reference, and DM was calculated to be approximately 18% for the low methacrylated ELP and 33% for the high methacrylated ELP based on Equation (1). Meanwhile, ELP macromolecules with hydrophobic side chains (([VPGVG]_4_[IPGVG])_14_) could readily form hydrophobic pockets to avoid interactions with water molecules (Figure 1c). Such structural alterations induced by hydrophobic interactions are well‐established and have been shown to provide thermodynamic stability to the polymer in solution.^54^ On the other hand, Bio‐IL was synthesized through the reaction of acrylic acid and choline‐based bicarbonate. The ^1^H NMR spectra of the acrylated Bio‐IL showed the characteristic multiple peaks in the range of 5.3–6.3 ppm, confirming the acrylation of Bio‐IL (Figure 1d, highlighted in yellow). Also, the appearance of a sharp peak at *δ* ∼ 3.1 ppm corresponds to the three hydrogen atoms of the ammonium ion (Figure 1d, highlighted in green).

### 3.2 Synthesis and chemical characterization of the mELP/Bio‐IL hydrogel

**FIGURE 2:**
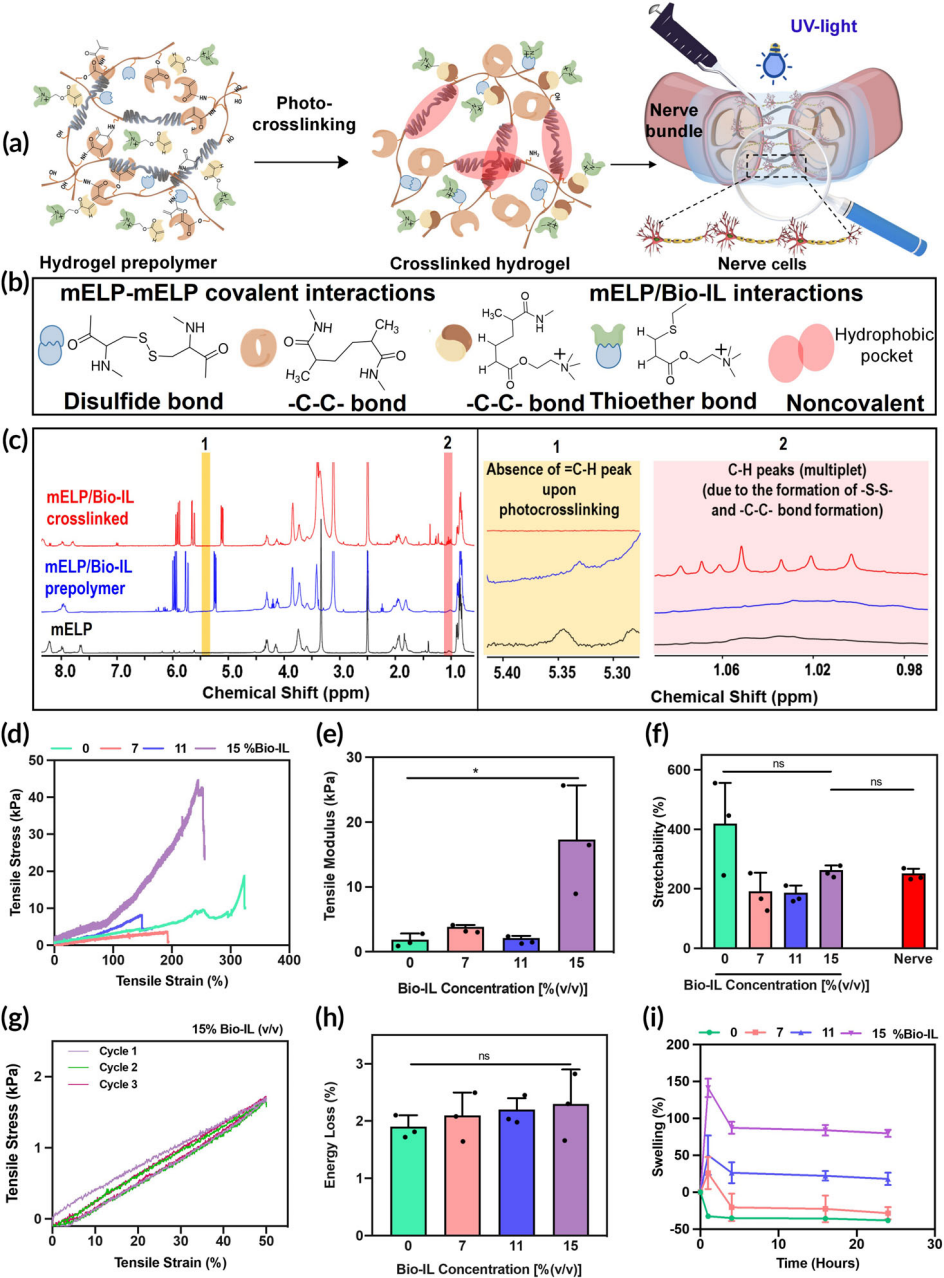
Synthesis and physicochemical properties of mELP/Bio‐IL hydrogel. (a) Schematic illustration of the crosslinking mechanism and the potential application for reconnection of injured nerve tissues; (b) chemical structures of covalent and noncovalent bonds formed after photocrosslinking process; (c) ^1^H NMR spectra of mELP and mELP/Bio‐IL before and after crosslinking; (d) representative tensile strain–stress curves, (e) tensile modulus, and (f) stretchability of mELP/Bio‐IL hydrogels prepared with 15% (w/v) mELP and different Bio‐IL concentrations compared to natural rat peripheral nerve; (g) representative cyclic tensile stress–strain curve for 3 cycles using mELP/Bio‐IL hydrogels prepared with 15% (w/v) mELP and 15% (v/v) Bio‐IL; (h) energy loss for mELP/Bio‐IL hydrogels after 8th tensile cyclic measurements; (i) swelling behavior of mELP/Bio‐IL hydrogels in DPBS solution at 37°C (*n* = 3; error bars indicate the standard error of the means, asterisks mark significance levels of *p < 0.05). ^1^H NMR, proton nuclear magnetic resonance; Bio‐IL, acrylated choline‐based bioionic liquid; ELP, elastin‐like polypeptide; mELP, methacrylated ELP.

Different concentrations of Bio‐IL (7%, 11%, and 15% (v/v)) were mixed with a 15% (w/v) mELP solution and photocrosslinked in the presence of Irgacure 2959 to engineer an elastic and conductive hydrogel for reconnection of injured nerve tissues (Figure 2a). A fixed concentration of 15% (w/v) mELP was used based on our previous study,^55^ confirming its suitability as a surgical sealant. The mELP/Bio‐IL hydrogel contained a variety of covalent and noncovalent interactions, leading to improved mechanochemical properties. For instance, mELP chains containing photoreactive methacrylate and cystine groups formed new covalent carbon–carbon (—C—C—) and disulfide (—S—S—) bonds, respectively, upon exposure to light. On the other hand, acrylate groups of Bio‐IL interacted with both methacrylate and cysteine groups of mELP upon light irradiation and formed various covalent bonds, as graphically illustrated in Figure 2b. In addition to these covalent bonding interactions, the mELP/Bio‐IL hydrogel network also contained noncovalent interactions through repetitive pentapeptide moieties made of amino acid building blocks with hydrophobic side chains (Figure 2b). These noncovalent interactions predominantly contribute to the stretchable characteristics of the engineered hydrogel. The crosslinked mELP/Bio‐IL hydrogel was chemically characterized using ^1^H NMR and compared with mELP and mELP/Bio‐IL prepolymer solutions. The results confirmed the formation of covalent networks between methacrylate groups in the crosslinked hydrogel, evident from the absence of peaks at 5.34–5.68 ppm, which were related to the methacrylate group of mELP (Figure 2c, region 1, highlighted in yellow). Meanwhile, the acrylate group of Bio‐IL also covalently interacted with the methacrylate and thiol groups of mELP, causing multiplet peaks in the region of 1–1.3 ppm (C—H bonds of —CH_2_ groups) (Figure S2). Furthermore, the multiplet peaks between 1.0 and 1.1 ppm confirmed the formation of the disulfide (—S—S—) bonds in the crosslinked mELP/Bio‐IL hydrogel network^56^ (Figure 2c, region 2, highlighted in pink).

### 3.3 Mechanical characterization

To repair injured tissues, it is essential to provide mechanical support while minimizing tissue‐material mechanical mismatches.^26^, ^28^ This strategy aims to reduce immune responses and minimize fibrotic tissue formation, thereby enhancing the functional recovery of the injured nerve tissue.^4^, ^57^ Furthermore, recent studies have shown that elastic properties of the hydrogel could provide mechanical cues and modulate cellular functions.^58^, ^59^

To assess the mechanical properties of the engineered mELP/Bio‐IL hydrogels, both tensile and cyclic tensile tests were conducted (Figure 2d–h).

It is important to note that, upon comprehensive mechanical analysis, we observed that mELP with lower DM showed low mechanical properties and limited stability (Figure S3). Therefore, we directed our analysis toward the combination of mELP with higher DM (33%) and different Bio‐IL concentrations. The hydrogels were composed of 15% (w/v) mELP with varying Bio‐IL concentrations (7%, 11%, and 15% (v/v)). Tensile tests on mELP/Bio‐IL hydrogels showed that the elastic modulus of engineered hydrogels could be tuned by varying Bio‐IL concentrations (Figure 2d,e). The elastic modulus of mELP/Bio‐IL hydrogels, with a total mELP concentration of 15% (w/v), remained nearly unchanged (between 2 and 4 kPa) after introducing different concentrations of Bio‐IL up to 11% (v/v). Further increase in Bio‐IL concentration to 15% (v/v) significantly increased the elastic modulus to 17.31 ± 8.37 kPa. Additionally, the toughness of mELP/Bio‐IL hydrogel containing 15% (v/v) Bio‐IL (9553.25 ± 1424 J/m^3^) was significantly greater than pure mELP scaffold (318.7 ± 68.5) (Figure S4). Extensibility within the range of 400% was also obtained for the pure mELP scaffold. However, with the addition of Bio‐IL, the stretchability decreased to ~200%, closely matching that of rat peripheral nerve tissue (Figure 2f). To test the resiliency of mELP/Bio‐IL hydrogels upon stretching, a cyclic tensile test was performed. The results confirmed the elastic nature of the hydrogel, exhibiting minimal energy loss (~2%) even after the 8th cycle (Figure 2g,h). Overall, the inclusion of ELP in mELP/Bio‐IL hydrogel contributes to the desired elasticity, stiffness, and stretchability necessary for fostering neural tissue growth, proliferation, and enhancing nerve regeneration in neural implants.^23^

### 3.4 In vitro swelling properties of mELP/Bio‐IL hydrogel

Swellability is a key factor in material design as it affects permeability, which in turn influences the diffusion of vital substances like nutrients, oxygen, and metabolic waste.^41^ Moreover, the mechano‐physical properties and the electroconductive properties of the hydrogel often change with swelling.^60^ Controlling hydrogel swelling becomes particularly vital to prevent expansion‐related pressure on confined nerves in restricted spaces.^61^ From a clinical perspective, implantable hydrogels should mitigate nerve compression caused by degradation‐induced swelling. Also, balancing degradation rates with nerve regeneration rates and limiting swelling are crucial to prevent compression of regenerating nerves, which could impede their growth and function.^61^ In light of this importance, the swellability of the mELP/Bio‐IL hydrogels, containing different concentrations of Bio‐IL, was investigated (Figure 2i). In this regard, weight measurements of the hydrogels at various time intervals over 24 h of incubation in DPBS at 37°C were conducted. Notably, pure mELP hydrogel, as a control, showed shrinkage due to the presence of hydrophobic residues in the ELP backbone. In contrast, mELP/Bio‐IL hydrogels demonstrated rapid swelling within the first hour, followed by a shrinking phase (within 4 h) and then a plateau phase. The initial swelling observed in Bio‐IL‐containing hydrogels, along with the escalated swelling rates in hydrogels with higher Bio‐IL concentrations, may be attributed to the charge‐induced swelling mechanism of Bio‐IL.^62^, ^63^ As a result, hydrogels containing Bio‐IL exhibited higher swelling rates compared to those without Bio‐IL, particularly at higher concentrations of Bio‐IL due to increased charge density. For instance, mELP/Bio‐IL hydrogel containing 7% (v/v) Bio‐IL swelled by approximately 25%, whereas the hydrogel with 15% (v/v) Bio‐IL showed a swelling ratio of around 145% within 2 h of incubation. Meanwhile, the shrinking of the hydrogels with time until 4 h could be related to the hydrophobic effect which stabilized the hydrogel structure by minimizing water‐polymer interactions. ELP‐based hydrogels are known to shrink and lose water due to hydrophobic molecular rearrangement at temperatures above their transition temperature.^42^, ^51^, ^55^ However, in equilibrium conditions, the overall swelling capability of the hydrogels increased with higher Bio‐IL concentrations. For example, mELP/Bio‐IL hydrogel with 11% (v/v) Bio‐IL exhibited swelling of approximately 30%, while hydrogel containing 15% (v/v) Bio‐IL showed swelling in the range of around 85%. This suggests that higher concentrations of Bio‐IL were associated with an increased swelling ratio when the hydrogels were in a stable, equilibrium state. Altogether, the swelling ratios obtained for all formulations fall within the clinically acceptable range (less than 300%), ensuring sufficient hydration and nutrient exchange while minimizing tissue damage or inflammation.^64^, ^65^, ^66^

### 3.5 Ex vivo adhesion test

**FIGURE 3:**
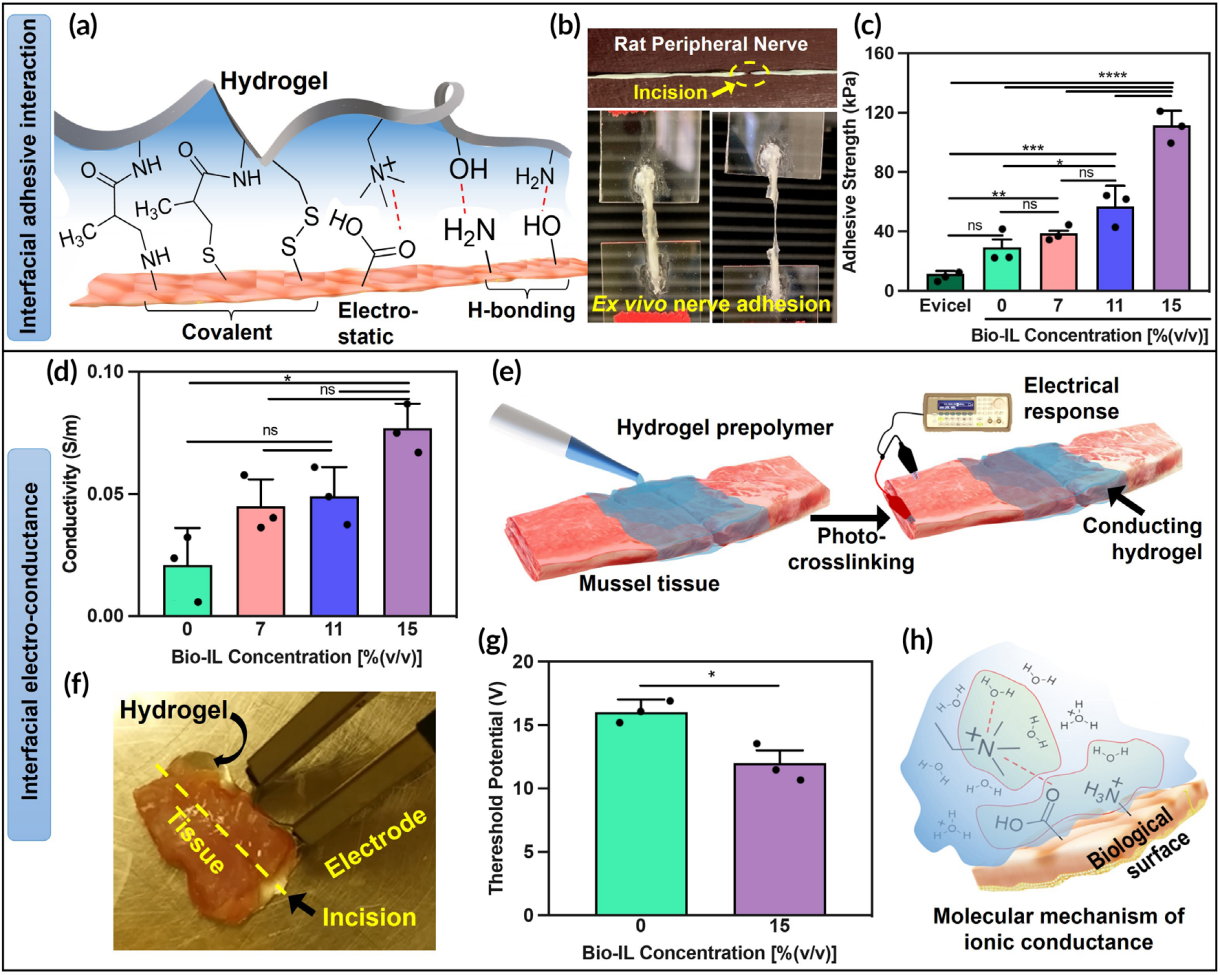
Adhesion assessment and in vitro and ex vivo conductivity measurements. (a) Schematics of chemical interactions at tissue and hydrogel interface; (b) representative picture of adhesion test between rat peripheral nerve and mELP/Bio‐IL hydrogel; (c) adhesive strength of different formulations of hydrogel with varying Bio‐IL concentration (0%, 7%, 11%, and 15% (v/v)) compared to commercially available fibrin glue (Evicel®); (d) in vitro conductivity of hydrogels with varying Bio‐IL concentration (0%, 7%, 11%, and 15% (v/v)) and 15% (w/v) mELP; (e) schematic of ex vivo conductivity measurement with rat abdominal tissue repaired/connected with mELP/Bio‐IL formed by using 15% (w/v) mELP, without Bio‐IL and with 15% (v/v) Bio‐IL to determine the threshold voltage needed to stimulate both sides of abdominal tissue; (f) a representative image of ex vivo conductivity test; (g) quantification of the threshold voltage of mELP/Bio‐IL hydrogel suggesting that hydrogels with 15% (v/v) Bio‐IL reduced the threshold potential for stimulation; (h) schematic showing the molecular mechanism of ionic conductance between hydrogel and tissue (*n* = 3; error bars indicate the standard error of the means, asterisks mark significance levels of *p < 0.05, **p < 0.01, ***p < 0.001, and ****p < 0.0001). Bio‐IL, acrylated choline‐based bioionic liquid; ELP, elastin‐like polypeptide; mELP, methacrylated ELP.

Successful repair and regeneration of nerve tissue necessitate the strong adhesion and durable retention of biomaterials at the injury site, capable of withstanding the stress associated with nerve tissue movement. The engineered mELP/Bio‐IL could establish diverse interactions, including H‐bonding, covalent interactions (Schiff base, thiolene), and electrostatic interactions with the native tissues (Figure 3a). To measure the adhesion strength, we performed an ex vivo adhesion test using explanted rat peripheral nerve tissue in a wound closure setup (Figure 3b,c). During the test, mELP/Bio‐IL hydrogels with different Bio‐IL concentrations (0%, 7%, 11%, and 15% (v/v)) were applied over the explanted nerve tissue surfaces (Figure 3b) and photocrosslinked to adhere through molecular interactions at the tissue‐material interface. Additionally, to compare the adhesive performance of the engineered hydrogel, commercial surgical glue, Evicel®, was also used. The adhesive strength of mELP/Bio‐IL hydrogels increased with higher Bio‐IL concentrations, likely due to enhanced electrostatic interactions at the tissue‐material interface (Figure 3c). For example, while pure mELP hydrogel (control) showed an adhesion strength in the range of 30–35 kPa, mELP/Bio‐IL hydrogels containing 11% and 15% (v/v) Bio‐IL showed tissue adhesion strengths of around 55 and 115 kPa, respectively. For all formulations, the engineered hydrogels demonstrated significantly improved adhesion performance compared to the existing literature^65^ and commercial surgical glue, Evicel®, which exhibited an adhesive strength of approximately 10 kPa toward the rat peripheral nerve tissue.

### 3.6 In vitro electrical conductivity of mELP/Bio‐IL hydrogels

In the successful design of nerve tissue adhesive reconnectors, the consideration of electrical conductivity is paramount for ensuring effective neural communication, tissue integration, and functional recovery.^67^ Neural signaling relies on electrical impulses, and adhesive sealants with adequate conductivity can facilitate the transmission of these signals, supporting normal nerve function.^68^ Moreover, the inclusion of conductivity promotes integration with surrounding native tissues, contributing to a more physiological environment.^69^ In addition, the electroactive properties of conductive sealants can further influence cell behavior, potentially aiding in cell growth and guiding neural tissue regeneration. In this regard, we designed electroconductive hydrogel by incorporating Bio‐ILs and evaluated the conductivity of the engineered mELP/Bio‐IL hydrogels by changing the Bio‐IL concentrations. The increased conductivity at higher Bio‐IL concentrations confirmed the molecular mechanism of conductance and the mobility of ions (ionic conductance) within the 3D network of the hydrogels. For instance, the conductivity of the pure mELP hydrogel was around 0.02 S/m, while the incorporation of 7% and 15% (v/v) Bio‐IL increased the conductivity to 0.05 and 0.08 S/m, respectively (Figure 3d). Given that the conductivity of nerve tissue is ∼0.08–1.3 S/m, our material, with conductivity levels similar to nerve tissue, is capable of effectively transmitting electrical signals to neurons.^70^ Unlike some methods that integrate conductive materials such as polyaniline, CNTs, GO, etc., into polymeric matrices to achieve higher conductivities, our approach mitigates potential issues such as reduced mechanical properties and increased cytotoxicity in vitro.^40^

### 3.7 Ex vivo electrical conductivity of mELP/Bio‐IL hydrogels

A conductive hydrogel can carry electrical stimulation from one side of the ruptured nerve tissue to the other side. To assess the ability of the mELP/Bio‐IL hydrogel to support nerve cells communication, an ex vivo electrical conductivity test using Wistar rat abdominal muscle tissue pieces was conducted (Figure 3e,f). Herein, two pieces of tissue were placed on a petri dish, with a 1 mm gap between them. The engineered hydrogel was then applied in the space between the tissues and photocrosslinked. Subsequently, a DC electrical pulse with varying voltages, ranging from low to high, was applied to one of the two pieces. The response of the other tissue was observed to determine the minimum threshold voltage required to introduce muscle tissue beating or contraction. Muscle contraction was visually assessed on the opposite sample and the corresponding threshold voltage was recorded (see Video S1). The results showed that mELP/Bio‐IL hydrogels exhibited a lower threshold voltage (~12 V) for muscle beating compared to the pure mELP hydrogels (~16 V). This outcome underscores the role of Bio‐IL in reducing the threshold voltage required for transmitting stimulation and re‐establishing tissue connectivity (Figure 3g). Meanwhile, the adhesive characteristics of the engineered hydrogel could also have a significant role in tissue conductance as it can actively modulate the tissue‐material interfacial barrier through the covalent and noncovalent interactions presented in Figure 3h.

### 3.8 In vitro biocompatibility of mELP/Bio‐IL hydrogels

**FIGURE 4:**
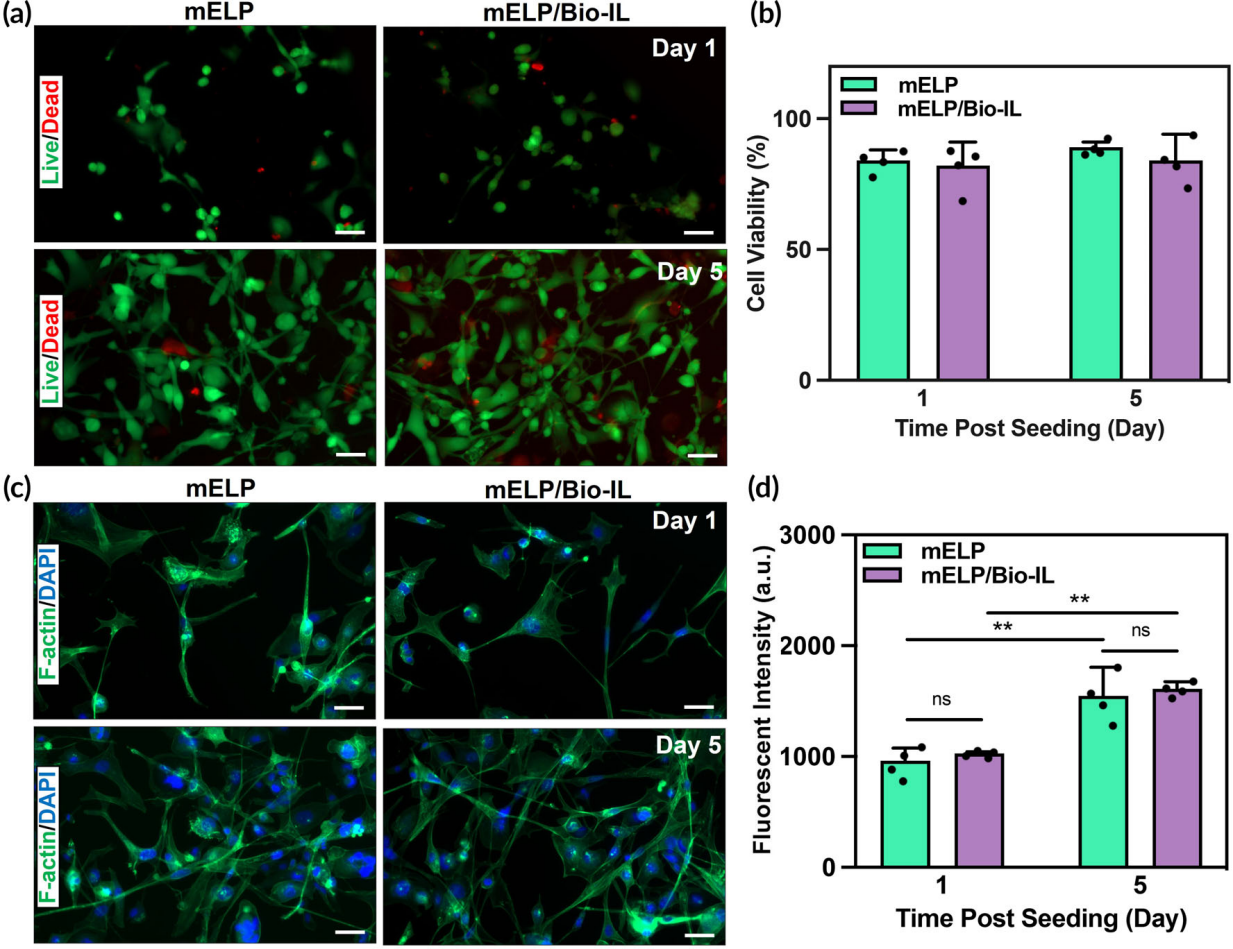
In vitro biocompatibility of mELP/Bio‐IL hydrogels. (a) Representative live/dead images of MO3.13 cells seeded after 1 and 5 days post‐seeding; (b) quantification of cell viability at days 1 and 5 post‐seeding; (c) representative F‐actin/DAPI stained images of the MO3.13 cells after 1 and 5 days post‐seeding; (d) quantification of metabolic activity, relative fluorescence units, using a PrestoBlue assay at days 1 and 5 post‐seeding. 15% (w/v) mELP and 15% (v/v) Bio‐IL were used (*n* = 4; scale bars = 100 μm; error bars indicate standard error of the means, asterisks mark significance levels of **p < 0.01). Bio‐IL, acrylated choline‐based bioionic liquid; DAPI, 4′,6‐diamidino‐2‐phenylindole; ELP, elastin‐like polypeptide; mELP, methacrylated ELP; MO3.13, human oligodendrocytic.

To evaluate the biocompatibility of the mELP/Bio‐IL hydrogel, MO3.13 cells were seeded at the bottom of transwell permeable supports with crosslinked mELP/Bio‐IL and pure mELP hydrogels placed in the transwell inserts, followed by incubation for 5 days (Figure 4a). Fluorescence images obtained from the stained samples using a live/dead kit exhibited that the majority of cells remained viable in contact with both mELP/Bio‐IL and mELP hydrogels. Also, the quantitative cell viability data at days 1 and 5 post‐seeding revealed excellent cell survival over the culture with >80% cell viability (Figure 4b). The F‐actin/DAPI stained exhibited proliferation and spreading of MO3.13 cells in contact with both mELP/Bio‐IL and mELP hydrogels up to day 5 post‐seeding (Figure 4c). Furthermore, the metabolic activity of the cells was assessed using a PrestoBlue cell viability reagent (Figure 4d), showing a rapid increase in the metabolic activity of MO3.13 cells for both hydrogel formulations up to 5 days post‐seeding. These studies together demonstrated the in vitro biocompatibility of mELP/Bio‐IL hydrogels, highlighting their potential for peripheral nerve tissue reconnection and repair.

### 3.9 In vivo biocompatibility and biodegradation of mELP/Bio‐IL hydrogels

**FIGURE 5:**
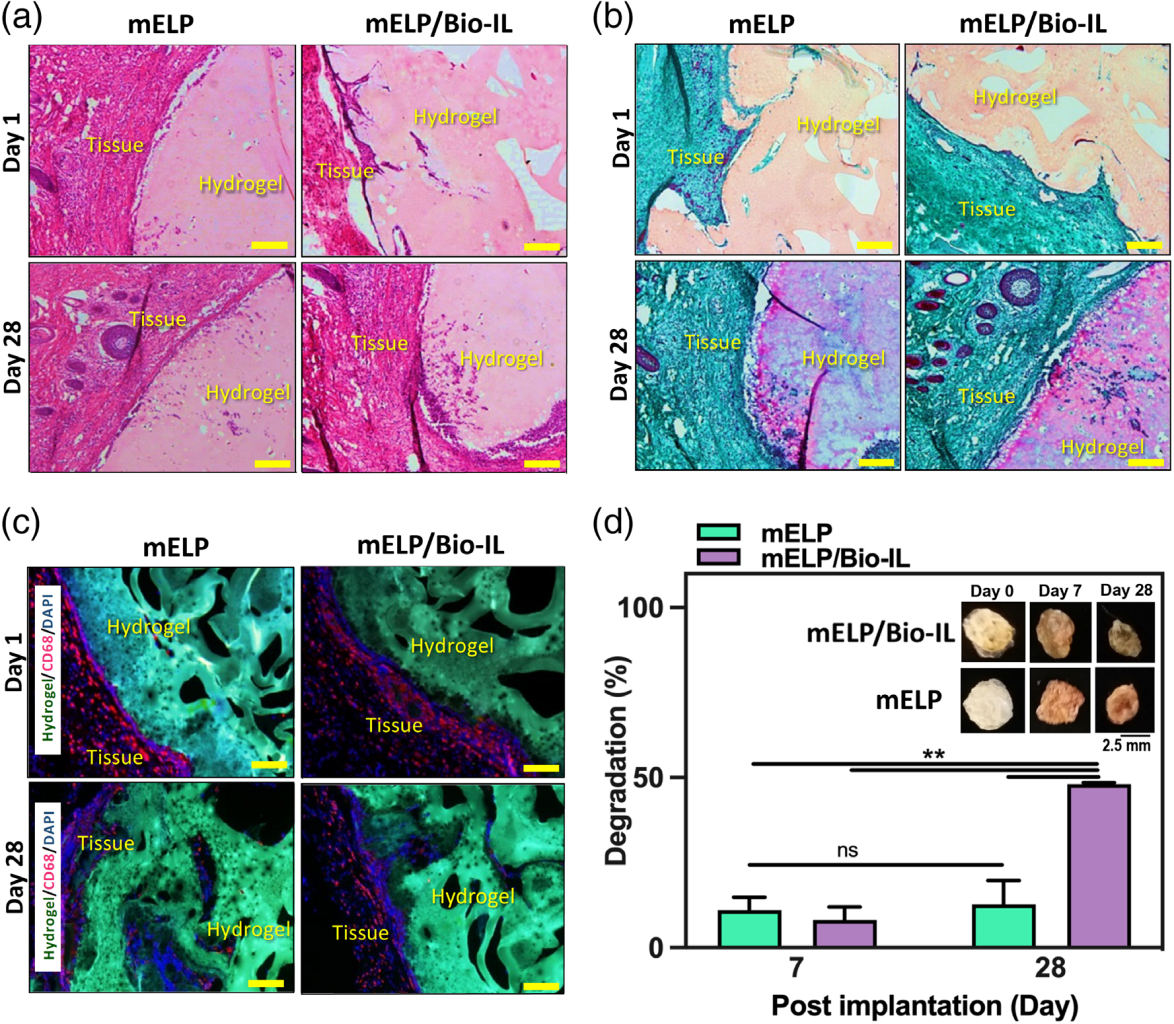
In vivo biocompatibility of mELP and mELP/Bio‐IL hydrogels using a rat subcutaneous model. (a) H&E staining; (b) MT staining; (c) fluorescent IHC staining of mELP and mELP/Bio‐IL hydrogels sections (hydrogels with the surrounding tissue) after 7, and 28 days of implantation; (d) quantitative in vivo biodegradation of hydrogels on days 7, and 28, Inset representative images of the hydrogels before implantation (day 0), and on days 7, and 28 post‐implantation (*n* = 2; scale bars = 100 μm; error bars indicate standard error of the means, asterisks mark significance levels of **p < 0.01). Bio‐IL, acrylated choline‐based bioionic liquid; DAPI, 4′,6‐diamidino‐2‐phenylindole; ELP, elastin‐like polypeptide; H&E, Hematoxylin and eosin; IHC, immunohistochemistry; mELP, methacrylated ELP; MT, Masson's trichrome.

We used a rat subcutaneous animal model to evaluate the in vivo biocompatibility of mELP and mELP/Bio‐IL hydrogels. The representative micrographs from H&E (Figure 5a) as well as MT (Figure 5b) staining displayed a significant amount of cell infiltration in both mELP/Bio‐IL and mELP hydrogels 28 days post‐implantation. This confirmed the biocompatibility of the hydrogels, which resulted in tissue ingrowth and integrity with the host tissue upon biodegradation. As shown in Figure 5b no significant fibrosis was detected. In addition, IF analysis of subcutaneously implanted hydrogels demonstrated macrophage (CD68) presence at day 7, which was reduced at day 28 (Figure 5c).

Furthermore, the in vivo biodegradation of the engineered hydrogels was evaluated by measuring the changes in the weight of the lyophilized hydrogels before implantation and at days 7 and 28 post‐implantation.^71^ Figure 5d shows the images of mELP/Bio‐IL and mELP hydrogels before implantation (day 0) and on days 7 and 28 post‐implantation. The change in size over time exhibited the biodegradation of the implanted samples. Also, changes in the color of the explanted mELP/Bio‐IL and mELP hydrogels could be attributed to tissue ingrowth during hydrogel degradation. Quantitatively, after 28 days post‐surgery, mELP/Bio‐IL hydrogels showed a degradation of 48.1 ± 0.3%, while the control group (mELP) hydrogel exhibited no significant weight difference (Figure 5d). This is consistent with our previous studies on elastin‐based biomaterials which showed slower in vivo degradation rate as compared to other protein‐based adhesives.^46^ In addition, faster in vivo degradation of mELP/Bio‐IL compared to mELP hydrogels could be due to their higher swelling ratio, which could facilitate water penetration in the hydrogel and cause rapid degradability.

While our pilot study confirmed the in vivo biocompatibility of the designed nerve glue with a limited number of rats, future investigations should be conducted with a larger sample size to explore the long‐term biocompatibility and degradation rate of the hydrogel. In addition, future in vivo testing should consider sex as a biological variable and its potential impact on result interpretation or translation.

## 4 CONCLUSION

In this study, we have successfully developed a new conductive and highly stretchable bioadhesive with the potential for the repair and sealing of nerve injury. The hydrogel is versatile due to its adaptability for repairing various types of soft tissues, achieved by fine‐tuning its physical, mechanical, and electrical properties. The hydrogel was made of ELP and Bio‐IL. The incorporation of Bio‐IL effectively introduced ionic conductivity into a stretchable mELP matrix. The resulting hydrogel exhibited improved conductivity, stretchability, adhesiveness, swelling capacity, and biodegradability. The synthesized hydrogel exhibited conductivity between 0.02 and 0.08 S/m and a stretchability of more than 200%. Notably, the elastic modulus of these adhesive hydrogels aligned closely with that of normal soft tissue, ranging from 1.8 to 15 kPa. Furthermore, our hydrogel demonstrated robust ex vivo adhesive strength to rat peripheral nerves when compared with a commercially available fibrin‐based adhesive, Evicel®. In vitro cell studies using MO3.13 cells revealed over 80% cell viability and increased cell proliferation. In vivo subcutaneous implantation results also demonstrated the biodegradability of the hydrogel, with minimal inflammatory response. These findings position our hydrogel as a viable candidate for nerve tissue repair, holding significant potential for applications in other internal organs that require conductivity, such as heart tissue. Our future work will focus on performing rigorous testing on peripheral nerve injury models in rats, like sciatic nerve defects, to assess the translational efficacy of the hydrogel for repairing and sealing nerve injuries.

## AUTHOR CONTRIBUTIONS

**Jharana Dhal:** Conceptualization; data curation; formal analysis; investigation; methodology; visualization; writing – original draft. **Mahsa Ghovvati:** Conceptualization; data curation; formal analysis; investigation; methodology; visualization; writing – original draft; writing – review and editing. **Avijit Baidya:** Data curation; formal analysis; visualization; writing – review and editing. **Ronak Afshari:** Formal analysis; validation; visualization; writing – review and editing. **Curtis L. Cetrulo:** Conceptualization; supervision; writing – review and editing. **Reza Abdi:** Conceptualization; supervision; writing – review and editing. **Nasim Annabi:** Conceptualization; funding acquisition; investigation; methodology; project administration; resources; supervision; validation; writing – original draft; writing – review and editing.

## CONFLICT OF INTEREST STATEMENT

Dr. Nasim Annabi holds equity in GelMEDIX Inc. The remaining authors declare no competing financial interest.

## Supporting information


**FIGURE S1:** ^1^H NMR spectra of ELP and mELP with low and high degrees of methacrylation synthesized with different concentrations of methacrylic anhydride (7.5 and 15% (v/v)). ^1^H NMR, proton nuclear magnetic resonance; DM, degree of methacrylation; ELP, elastin‐like polypeptide; mELP, methacrylated ELP.
**FIGURE S2.** Expanded ^1^H NMR spectra of mELP and mELP/Bio‐IL before and after crosslinking. ^1^H NMR, proton nuclear magnetic resonance; Bio‐IL, acrylated choline‐based bioionic liquid; ELP, elastin‐like polypeptide; mELP, methacrylated ELP.
**FIGURE S3.** Tensile modulus of mELP/Bio‐IL hydrogels prepared with high and low methacrylation degrees of mELP and different Bio‐IL concentrations. Bio‐IL, acrylated choline‐based bioionic liquid; ELP, elastin‐like polypeptide; mELP, methacrylated ELP.
**FIGURE S4.** Toughness of the mELP/Bio‐IL hydrogels (hydrogels were prepared with 15% (w/v) mELP; *n* = 3; error bars indicate the standard error of the means, asterisks mark significance levels of *p < 0.05, **p < 0.01). Bio‐IL, acrylated choline‐based bioionic liquid; ELP, elastin‐like polypeptide; mELP, methacrylated ELP.

**VIDEO S1:** Ex vivo evaluation of mELP/Bio‐IL hydrogel capability to restore propagation across explanted rat muscle tissue.

## Data Availability

Data available on request from the authors.
