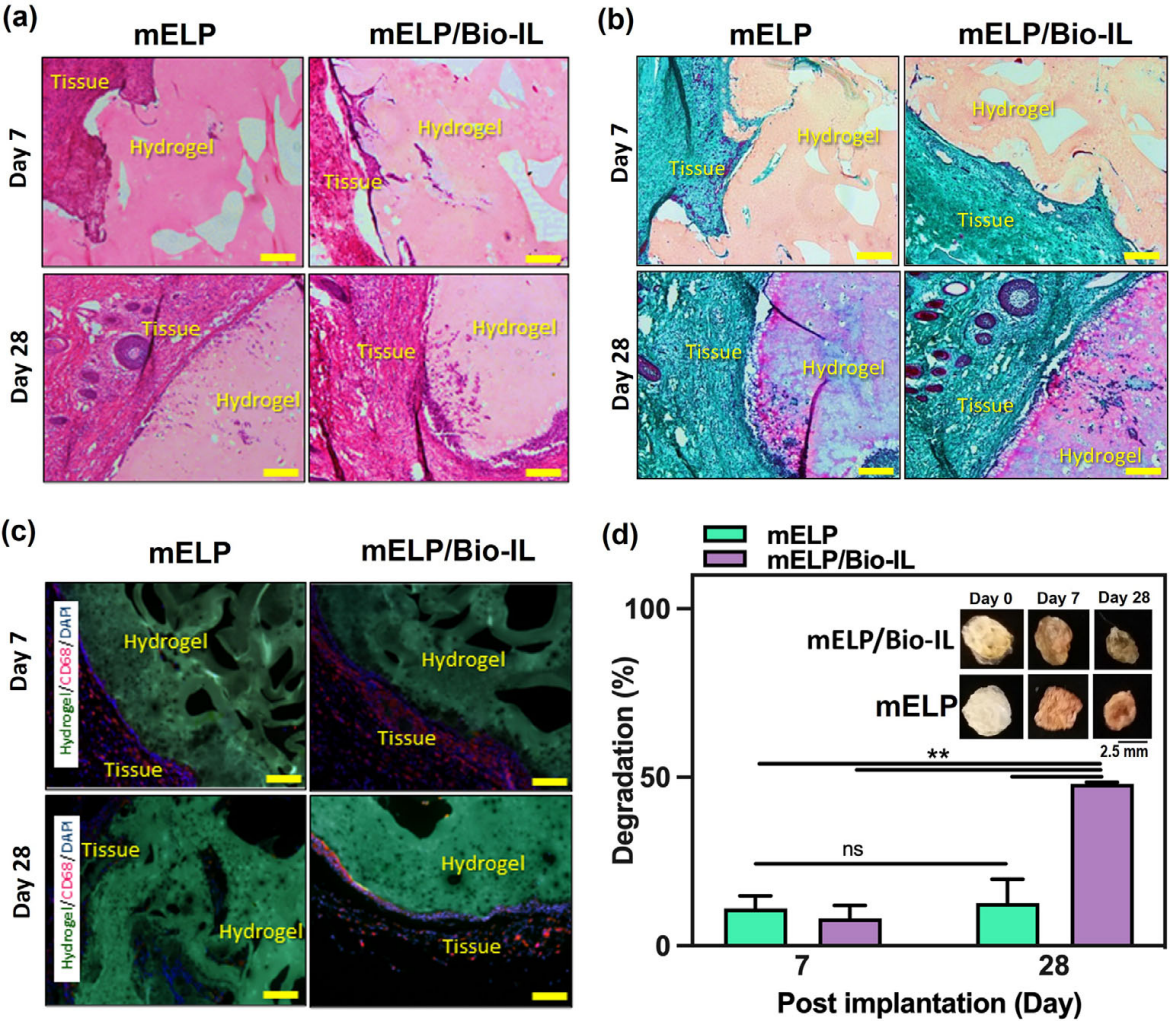# Correction to “A stretchable, electroconductive tissue adhesive for the treatment of neural injury”

**DOI:** 10.1002/btm2.10759

**Published:** 2025-03-19

Dhal J, Ghovvati M, Baidya A, et al. A stretchable, electroconductive tissue adhesive for the treatment of neural injury. *Bioeng Transl Med*. 2024;9(5):e10667. doi:10.1002/btm2.10667

An error occurred in the placement of the two representative images in Figure 5a (mELP on Day 7) and Figure 5c (mELP/Bio‐IL on Day 28) during the figure assembly process. Additionally, a labeling error was identified in the time point annotation. The corrected version of the images, derived from the original data, are incorporated in the figure shown below. This correction does not impact the data interpretation and conclusions of the study. The authors would like to apologize for this error.