# Supplementary material for: A stretchable, electroconductive tissue adhesive for the treatment of neural injury
Source: Bioeng Transl Med. 2024 May 3;9(5):e10667. doi: 10.1002/btm2.10667 (PMC11561837; doi:10.1002/btm2.10667)
Supplement: Supplementary file 1 — FIGURE S1: 1H NMR spectra of ELP and mELP with low and high degrees of methacrylation synthesized with different concentrations of methacrylic anhydride (7.5 and 15% (v/v)). 1H NMR, proton nuclear magnetic resonance; DM, degree of methacrylation; ELP, elastin‐like polypeptide; mELP, methacrylated ELP. FIGURE S2. Expanded 1H NMR spectra of mELP and mELP/Bio‐IL before and after crosslinking. 1H NMR, proton nuclear magnetic resonance; Bio‐IL, acrylated choline‐based bioionic liquid; ELP, elastin‐like polypeptide; mELP, methacrylated ELP. FIGURE S3. Tensile modulus of mELP/Bio‐IL hydrogels prepared with high and low methacrylation degrees of mELP and different Bio‐IL concentrations. Bio‐IL, acrylated choline‐based bioionic liquid; ELP, elastin‐like polypeptide; mELP, methacrylated ELP. FIGURE S4. Toughness of the mELP/Bio‐IL hydrogels (hydrogels were prepared with 15% (w/v) mELP; n = 3; error bars indicate the standard error of the means, asterisks mark significance levels of *p < 0.05, **p < 0.01). Bio‐IL, acrylated choline‐based bioionic liquid; ELP, elastin‐like polypeptide; mELP, methacrylated ELP. [file BTM2-9-e10667-s001.docx]

**Supporting Information**

**A Stretchable, electroconductive tissue adhesive for the treatment of neural injury**

Jharana Dhal^a^, Mahsa Ghovvati^a,b^, Avijit Baidya^a^, Ronak Afshari^a^, Curtis L.Cetrulo Jr^c^, Reza Abdi^d^, Nasim Annabi^a,e*^

^a^Department of Chemical and Biomolecular Engineering, University of California – Los Angeles, Los Angeles, CA 90095, USA

^b^Department of Radiological Sciences, David Geffen School of Medicine, University of California – Los Angeles, Los Angeles, CA 90095, USA

^c^Division of Plastic Surgery, Massachusetts General Hospital, Boston, MA, USA

^d^Transplantation Research Center, Nephrology Division, Brigham and Women’s Hospital, Boston, MA, USA

^e^Department of Bioengineering, University of California – Los Angeles, Los Angeles, CA 90095, USA

ORCID: Mahsa Ghovvati (0000-0002-0608-5768), Avijit Baidya (0000-0001-5215-2856), Ronak Afshari (0000-0003-4688-4406), Curtis L.Cetrulo Jr (0000-0001-7080-3894), Reza Abdi (0000-0002-8875-4469), Nasim Annabi (0000-0003-1879-1202)

***Corresponding Author:** Dr. Nasim Annabi: [nannabi@ucla.edu](mailto:nannabi@ucla.edu)

^^

**Figure S1**- ^1^H NMR spectra of ELP and mELP with low and high degrees of methacrylation synthesized with different concentrations of methacrylic anhydride (7.5 and 15% (v/v)). *Abbreviations*: ^1^H NMR: proton nuclear magnetic resonance; ELP: elastin-like polypeptide; mELP: methacrylated ELP; DM: degree of methacrylation.

**Figure S2**- Expanded ^1^H NMR spectra of mELP and mELP/Bio-IL before and after crosslinking. *Abbreviations*: ^1^H NMR: proton nuclear magnetic resonance; Bio-IL: acrylated choline-based bioionic liquid; ELP: elastin-like polypeptide; mELP: methacrylated ELP.

^
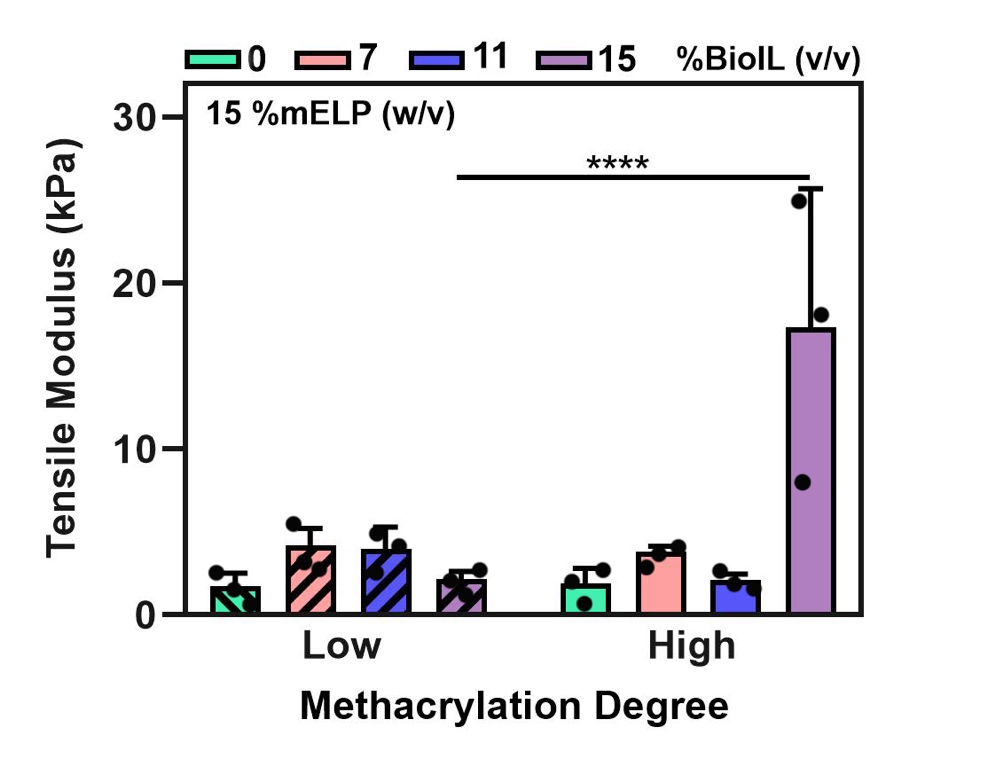
^

**Figure S3**- Tensile modulus of mELP/BioIL hydrogels prepared with high and low methacrylation degrees of mELP and different BioIL concentrations. *Abbreviations*: Bio-IL: acrylated choline-based bioionic liquid; ELP: elastin-like polypeptide; mELP: methacrylated ELP.


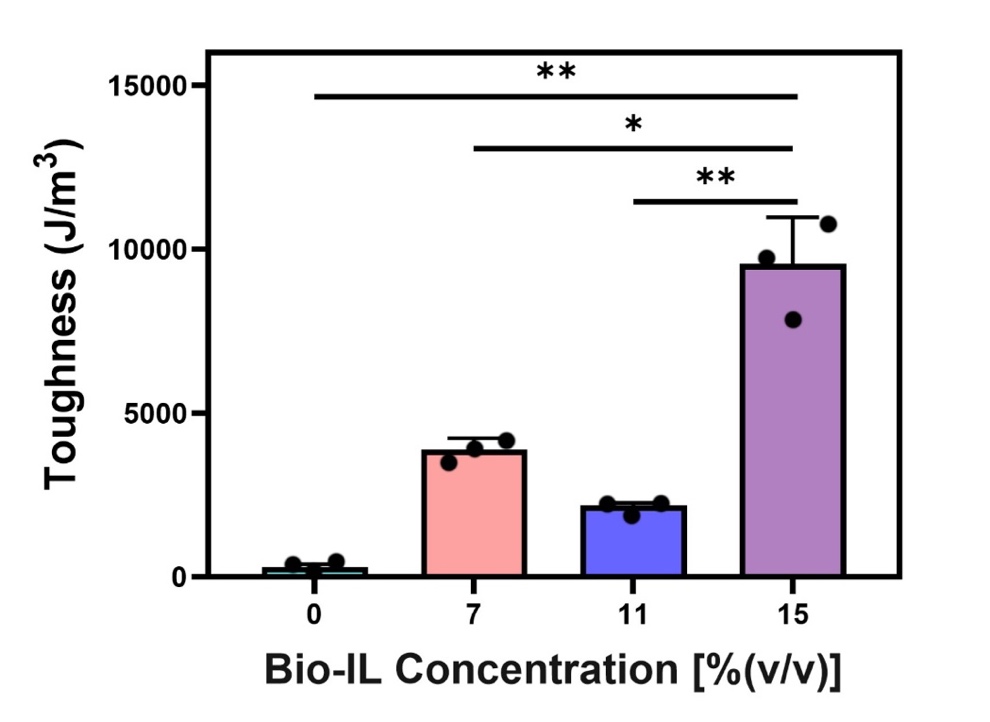


**Figure S4**- Toughness of the mELP/Bio-IL hydrogels (Hydrogels were prepared with 15% (w/v) mELP; n=3; error bars indicate the standard error of the means, asterisks mark significance levels of *p˂0.05, **p˂0.01). *Abbreviations*: Bio-IL: acrylated choline-based bioionic liquid; ELP: elastin-like polypeptide; mELP: methacrylated ELP.
